# “I clearly have some wiring that's not quite right” how general practitioners with lived experience of burnout describe personal factors related to their burnout: a qualitative study using in-depth interviews

**DOI:** 10.1093/fampra/cmag012

**Published:** 2026-03-31

**Authors:** I O Whitehead, B Hanratty

**Affiliations:** Population Health Sciences Institute, Faculty of Medical Sciences, Newcastle University, Newcastle upon Tyne NE4 5PL, United Kingdom; Population Health Sciences Institute, Faculty of Medical Sciences, Newcastle University, Newcastle upon Tyne NE4 5PL, United Kingdom

**Keywords:** burnout, general practice, primary care, spiritual health, neurodiversity, trauma

## Abstract

**Objective:**

To explore the personal factors that burned out GPs feel could have predisposed them to their burnout.

**Design:**

Secondary analysis of in-depth qualitative interviews with 16 UK GPs with “lived experience” of burnout.

**Setting:**

United Kingdom Primary Care.

**Results:**

Seven male and nine female GPs described the personal characteristics and experiences they believed contributed to their burnout. Themes identified were perfectionism; neurodivergence specifically likely autism; past trauma, including childhood trauma; and a sense of “imposter syndrome”. Participants were articulate and self-aware in the multifactorial causes of burnout, as well as their personal strengths. They identified longstanding traits that could increase vulnerability to burnout, they were often linked with empathy, conscientiousness, and commitment to patient care. Despite the challenges described, participants demonstrated resilience and a strong vocational identity as GPs.

**Conclusions:**

Burnout among GPs arises from complex interactions between personal and systemic factors. Traits such as perfectionism, neurodivergence, and prior trauma were described by participants as shaping vulnerability to burnout, while also underpinning the empathy and dedication valued in general practice. Recognizing and supporting, rather than pathologising, these vulnerabilities could help reduce stigma and enable earlier, more effective support. A systems-based approach that acknowledges the humanity of doctors is essential to sustaining a healthy, compassionate workforce. Further research should explore how best to support neurodivergent GPs and those with histories of trauma.

Key message

- While GP burnout is shaped by multiple interacting factors, some GPs describe personal characteristics and experiences that they perceive as contributing to their vulnerability to burnout. Recognising and understanding these perceived vulnerabilities, rather than viewing them as deficits, may help inform more supportive approaches earlier in a GP's career.

## Introduction

Burnout among UK General Practitioners (GPs) has become a major occupational health concern, reflecting a global trend of rising stress and exhaustion in primary care [1–3]. Multiple surveys have shown high levels of burnout in UK GPs [4, 5]. This has significant implications for practitioner wellbeing, the stability of the workforce, and quality of patient care [5–9].

Conceptually, burnout is viewed as an occupationally induced state of emotional depletion, detachment from professional role, and reduced sense of efficacy [10]. Its impact extends across psychological, physical, and social domains of health [2, 6, 11, 12]. Although widely recognized, the mechanisms leading to burnout are multifaceted and not yet fully understood [13–16]. Most existing research has focused on organizational and systemic stressors [2], whereas far less attention has been directed toward the individual dispositions and life experiences that may heighten vulnerability to burnout among doctors.

Traditional biomedical perspectives, while dominant in medicine, provide only a partial explanation for such complex human experiences [17–19]. Quantitative investigations have described prevalence and external correlates [2, 3, 20–23], and commentaries have debated structural causes; [24–28] however, the internal and personal dimensions of vulnerability remain largely unexplored. Identifying modifiable personal risk factors, beyond immutable demographics such as age, gender, or marital status, could support more tailored prevention strategies.

This study draws on a large dataset of qualitative interviews with GPs who self-identified as having experienced burnout, to understand how GPs experience burnout and their wider spiritual health (as defined by GPs) [29], and any interactions between these two concepts. In this study, lived experience refers to clinicians' reflective accounts of having experienced burnout over time, rather than contemporaneous symptom severity at the time of interview. We undertook a secondary analysis of the interview data to explore the personal characteristics, experiences, and vulnerabilities that participants perceived as contributing to their own burnout, with the aim of informing both individual awareness and system-level support, prompted by a question regarding predisposing factors to burnout was added to the interview guide during our public engagement work.

## Methods

This study was grounded within a pragmatic research paradigm, which allows investigation of topics such as the experience of burnout and spiritual health, and applications of that knowledge. The qualitative methods used are particularly suited to addressing such questions, as it allows in-depth exploration of meaning, interpretation, and experience rather than measurement or population-level inference.

Interviewees were recruited from GPs who had completed a survey on burnout and spiritual health [4, 29], and volunteered for an interview.

Survey recruitment was via email invitation and social media. Participants needed to have worked in UK general practice and have personal experience of burnout in the past. Purposive sampling was used to obtain a diverse group of GPs in terms of early, mid and late career, geography, and ethnic origin. Participants were purposively recruited on the basis of self-identified lived experience of burnout and sufficient reflective distance from the most acute phase to enable ethical participation and in-depth, meaningful reflection aligned with the study aims. The survey had 1318 respondents, 25 volunteered for interview, 16 took part. Interviews continued until thematic saturation was reached. A GP researcher (IOW) conducted in depth video interviews, using Zoom [30], with 16 GPs in the UK between September 2021 and February 2022. An interview guide was developed (Supplement S1), however interviewees were given space to tell their stories. Interviews lasted from 49 to 75 min. Consent was obtained to record the interview, and for subsequent dissemination of the findings. (Risk assessment in Supplement S2) Audio recordings were pseudonymized, then auto-transcribed [31], and corrected by the researcher. Interviews were conducted by a GP researcher without personal lived experience of burnout, using reflexive practice and a reflective diary to document assumptions and analytic decisions. In-depth interviews prioritized participants' narratives, with clinical listening skills used to facilitate open exploration of experience. A reflexive thematic analysis was conducted in NVivo [32, 33]. This involved familiarization with the data, coding, development of themes, and report writing [32]. The topic guide was revised after input from the public and the question of “why are GPs burning out?” was directly raised by a patient and public involvement (PPI) group (Voice, https://voice-global.org/), and a further question regarding personal predisposing factors was added after PPI work. The Copenhagen Burnout Inventory was used in the post interview survey, as it includes a personal burnout subscale that aligned with our focus on personal predisposing factors, and it has good evidence of reliability and validity across healthcare settings. Burnout scores were used descriptively to contextualize narratives and did not inform inclusion or the qualitative analysis

This is a secondary analysis of this large dataset, focussing on the data given about personal predisposing factors to burnout. Participants were invited to comment on a version of this article, for member checking. Autistic doctors were asked to comment to ensure neuroaffirmative language was used where neurodivergence is discussed. The protocol is contained in Supplement S3.

## Results and analysis

Sixteen GPs were interviewed. Table 1 summarizes gender, and personal burnout scores at the time of the interview. Six participants were experiencing moderate personal burnout, and two severe personal burnout. (Table 1) One participant trained outside the UK, and 13 participants were of white ethnicity. Other demographics were collected, but are not reported to avoid identification.

**Table 1:** Participant characteristics and total personal burnout score.

Participant	Gender*	Total personal burnout (from copenhagen burnout inventory) [34]** postinterview
**1**	Man	66.67
**2**	Man	45.83
**3**	Man	41.67
**4**	Woman	91.67
**5**	Woman	37.50
**6**	Woman	54.17
**7**	Woman	16.67
**8**	Woman	58.33
**9**	Man	62.50
**10**	Woman	70.83
**11**	Woman	37.50
**12**	Woman	54.17
**13**	Woman	79.17
**14**	Man	29.17
**15**	Man	Not given
**16**	Man	Not given

^*^Gender was recorded as per General Medical Council registration data.

^**^The Copenhagen burnout inventory consists of nine items across three burnout domains, personal, work and patient. Moderate burnout is suggested by scores of 50–74, high burnout scores 75–99, and 100+ for severe burnout.

**Figure 1:**
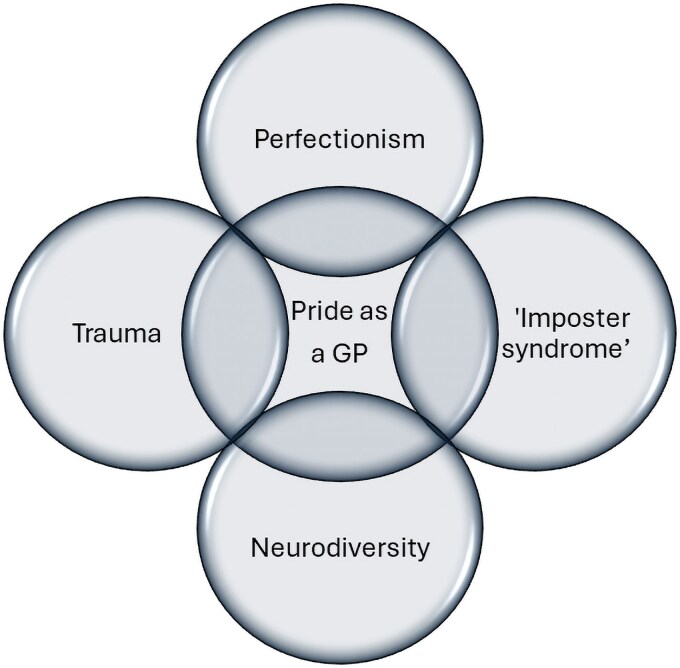
Intersecting themes of predisposing factors to burnout in GPs. Venn diagram illustrating the overlapping factors identified in qualitative interviews with GPs. *Pride in being a GP* is positioned at the centre, reflecting its overarching identity despite the interaction of four surrounding themes: perfectionism, “imposter syndrome”, neurodiversity, and trauma that were described as contributing to burnout. The areas of overlap indicate the co-occurrence and mutual reinforcement of these experiences in participants' narratives, rather than discrete or independent constructs. The figure conveys how professional pride was maintained through, and not despite, these factors.

Throughout the interviews, GPs described multiple facets of their burnout experience. The data presented here consider themes related to the question, “Are there personal predisposing risk factors to GP burnout?” and are summarized in Fig. 1.

### Perfectionism and other personality traits

#### Perfectionism

Through these interviews, a noticeable phenomenon of perfectionism and competitive nature was evident the interviewees were not the type to simply attend a yoga class a number had trained to become the yoga teacher, even in one case, travelling abroad to do so. Participants demonstrated this drive to the top in many areas: work, sport, and religion (becoming a leader). One participant described her attitude towards work as like an addiction, but recognized a similar addiction to fitness as well. Another participant described their interest in sport as “unhealthy”.

“The quickest swim time, I must have the best bike time” (GP2)

This was coupled with a feeling that others would not do things to the correct standard. Partly this was related to the nature of the partnership model, and a belief in the continuity of care. However when close to burnout, participants appeared to have a counter-productive belief that only they could do the job to the best standard. Being thorough was a source of pride, although acknowledged as a limitation by one participant who was unable to return to 10 minute consultations. Some participants owned the personality trait, with one describing themselves as a “completer finisher extraordinaire!” (GP 6)

“I'm very much a completer finisher, so I would have worked really long day so I could leave without that feeling that I had lots that I hadn't done.” (GP 5)

A strong vocation to “fix” others, a strong sense of ethical code, also appeared to contribute to burnout:

“And actually that part of the burnout was recognizing that actually, I keep trying to fix people and that's who GPs are.” (GP 8)

#### Imposter syndrome

In contrast to those who felt that if they needed the job doing, they should do it themselves, four participants described clearly a feeling of inferiority, a perception of not being as good as their peers, a fear of being found out, which they termed “Imposter Syndrome”. This appeared to be as damaging a drive as the perfectionism and competitive traits. The experience of burnout appeared to add confirmation bias to their sense of inferiority, and described how they addressed this when discussing their recovery from burnout.

“I realised that I actually had a sense of I’m a bad person and everyone… I’m not a good person, and so I kind of have to make sure no one finds out about that, so I have to compensate for that´.” (GP 7)

“I was really kind of sure that I wasn't doing this right. There was something about me I’ve got this job wrong. And I needed to do better. Work smarter.” (GP 11)

### Neurodiversity

One participant shared their diagnosis of autism, and two more shared that they self-identify as autistic. The autistic traits they described were also evident in two further interviews, of specific interests, social struggles and sensory sensitivities. Participants recognized the skills that autism gives, but also the limitations:

“My brain is really good at “give me a problem and I'll sort it out,” but land me with three or four things in my executive function at the same time and it just gets scrambled and it doesn't work well for me.” (GP 9)

“You know I’ve been one of the popular doctors with patients and … you know you kind of hear over and over, you know, “the first time I’ve ever been heard” you know so that you have people have different skill sets and these particular skill sets are needed, but they probably also put you at higher risk of burnout.” (GP 12)

The diagnosed participant struggled to get adequate reasonable adjustments, despite the Equality Act 2010:

“My partnership weren't very accommodating to any reasonable adjustments for autism, not really interested in that.” (GP 9)

Participants who were aware of their autistic traits described a belief that autism is under-recognized among doctors, particularly among those who experience burnout, drawing on their understanding that burnout is also commonly discussed within autistic communities. One participant suggested earlier screening during medical training for challenges such as autism, while emphasizing that any such approach would need to recognize that autistic traits are not purely negative.

### Trauma

Participants discussed various traumas that preceded their burnout, as well as the trauma of burnout itself. Past work itself was described as a traumatic experience, for example being a junior doctor in “the bad old days,” working in challenging circumstances abroad (two participants), or being in an unsupportive workplace.

“I still feel very, very bitter about how I was treated in the early 90 s, but no one cared and that was how, you know, the same old thing, and I don't think they actually believe us.” (GP 4)

Childhood trauma, or adverse childhood experiences (ACEs) were shared as a predisposing factor for burnout. One participant (GP 10) describes her childhood trauma as the “drive” to get out from home, and get through medical school, but also a vulnerability to burnout. Those who had survived trauma, and subsequently burned out, do not lack resilience. Rather, they may have demonstrated extraordinary resilience and drive.

“I don't think so much about resilience, I think, actually I’m quite resilient.” (GP 12)

Participants were open about their trauma, three having read extensively about ACEs, and self-reflective on how these early experiences shaped them, their medical careers, and their subsequent burnout.

“I've got quite a high ACE score as well. So I think possibly, like the burnout possibly came from a bit of adrenalisation, like just being in that fight or flight thing unknowingly.” (GP 10)

### Pride as a GP

While discussing their burnout, participants also talked about the personal traits that made them a good doctor a sense that “I’m good at what I do”. Despite much honesty and brutal self-reflection in the interviews, participants were clear that they were good doctors, even if that gift was in making the difficult decisions needed.

“I’m not brilliant in as in you know I’m not academic as in. not… you know just normal very normal average. Average person, but very dedicated to the profession.” (GP 4)

“I'm kind of probably the type of GP that likes to go the extra mile for probably everyone I see.” (GP 10)

This pride and sense of vocation was enough to bring many of the participants back to practice after their burnout, with a strong sense that their role as a GP was as part of them as other aspects of their personality.

## Discussion

### Summary of main findings

In this secondary analysis of interviews with UK GPs, participants identified perfectionism, imposter feelings, neurodiversity (often autism), and personal trauma as factors they perceived as increasing their personal risk of burnout. Importantly, participants also described pride in their work and a vocational identity as GPs, suggesting that these same traits may underpin qualities valued in practice.

A challenge placed by a member of the public in PPI work early in this research was the statement “I think it's great you’re doing this research we can find out which ones are likely to burnout and chuck them out early!” In this study, we have identified personality traits; perfectionism and competitive traits, imposter syndrome; and underlying factors such as neurodiversity and past trauma that predispose doctors to burnout. However, the participants retained a pride in their practice and their identity as GPs and doctors. While we can identify vulnerabilities in GPs which may be associated with burnout, these vulnerabilities could be the exact traits which make good GPs. The drive to be the best they can, whether driven by a tendency to perfectionism or a sense of imposter syndrome, is unsurprising in a population that has chosen a competitive career, that required high achievement, and high achievement is what the public expect of their GPs [35]. Patients want doctors who can communicate well, with kindness, empathy and who are clinically competent and thorough [36]. Autistic patients may find autistic doctors easier to communicate with [37]. Those with these desirable traits that predispose to higher detail orientation, empathy, and hard work may well be those with underlying neurodivergence, personalities, or backgrounds that have cultivated these traits. Perfectionism may create doctors with high standards, but vulnerabilities to inability to delegate. Trauma may create doctors with resilience, but an underlying vulnerability. A sense of “imposter syndrome” may create doctors who are humble and relatable, but may reduce confidence, and therefore competence. References to neurodivergence reflect participant-raised experiences and interpretations rather than epidemiological claims, and are presented in recognition that neurodivergence represents a meaningful minority within the workforce.

Patients want humane doctors [38] and with humaneness comes humanity, with factors which may predispose to burnout. Jung's “Wounded Healer” archetype [39] supports this entanglement of healing and healer. If suffering is universal, it is to be expected that doctors will also have experience of suffering [40]. Conchar *et al*. question whether care of staff at work is “occupational hygiene” or “dirty laundry”? [41] While some members of the public, like our PPI member, may view openness about the wounds of the healer to be “dirty laundry”, denial of the humanity of doctors could leave patients and doctors without care. Embracing the human and imperfect interactions between wounded healer and wounded patient may lead to greater healing for both parties.

### Strengths and limitations of this study

A strength of this study is the in depth interview method, and peer research, which allowed participants to provide valuable data around their personal experiences of burnout, and what they felt predisposed them personally to burnout. This is a qualitative study, investigating those personal predisposing factors burned out GPs themselves felt contributed to their experience, and does not mean that all GPs with these risk factors will be at risk of burnout.

As a peer researcher, the interviewer's professional background may have shaped data collection and interpretation; however, this positionality also facilitated rapport and depth of disclosure, which may have enriched the data.

Participants were a heterogeneous group, sampled to give a spectrum of experience rather than to reflect the general GP population, and therefore not representative of all GPs. The interviews produced rich data from early, mid and late career GPs, men and women, with a variety of socioeconomic backgrounds, ethnicities, and religions. The Copenhagen Burnout Inventory scores showed a range of current levels of burnout. Unfortunately, the lack of data from International Medical Graduates (IMGs) is a limitation of this study, and further research in this group is needed.

### Comparison with other literature

Personality traits such as perfectionism have previously been identified as potentially developing maladaptive qualities and fuelling burnout [42], however there can be both maladaptive perfectionism and beneficial conscientious striving [43]. Participants in our study described both elements: high standards and persistence (strivings), but also self-criticism and inability to delegate (concerns). Similarly, imposter syndrome has been linked with burnout, anxiety, and reduced job performance among clinicians [44]. Our data illustrate how these experiences manifest in GP practice, reinforcing the need for support even when such traits co-exist with professional competence.

There has been previous research on the effects of trauma within mental healthcare workers [41, 45] and social workers [45, 46]. However the evidence on whether this leaves workers more vulnerable, or acts as a “stress inoculation”, increasing resilience, is incomplete [41, 46]. The medical community is starting to consider the effects of ACEs and trauma on the physical and mental health of their patients [47]. To date, there is little evidence of meaningful reflection on how previous trauma could impact on practising doctors. There have been some studies quantifying ACEs in medical students [48]. There is little research into how ACEs may affect doctors in the longer term, or how doctors who experienced ACEs are affected by clinical practice and whether this differs from those with more childhood protective factors.

Autistic and neurodivergent doctors are becoming more connected and visible, embracing the strengths of neurodiversity [37, 49]. Studies in medical students have identified some stressors for autistic future doctors [50]. A study into self-recognition of autism in psychiatrists describes many of the barriers, but also the strengths that come from diagnosis and self-awareness [49]. A cross-sectional study of autistic doctors found 77% had considered suicide [51]. Despite the obvious importance, research with practising autistic GPs is sparse, and we know little about how their autism impacts on their practice and wellbeing.

The causes of GP burnout are certainly multifactorial [13–16]. While quantitative studies have identified associations between burnout and occupational, behavioural, socioeconomic [52], and some individual psychological characteristics [53], they offer limited insight into how clinicians themselves interpret intrinsic personal traits and life experiences in shaping vulnerability to burnout, which was the focus of this analysis. However, the lack of evidence on how predisposing psychological factors interact with other, (e.g. workplace), factors to precipitate burnout needs to be addressed [16].

## Conclusion

This study adds to understanding of GP burnout by providing in-depth insights into how some GPs interpret personal factors in relation to their own burnout experiences. With rising awareness and understanding of the impacts of trauma, ACEs [54], and neurodiversity in the wider public [55–57], this study shows the importance of recognizing these issues within the medical workforce. Our understanding of burnout related factors needs to extend beyond the demographic characteristics explored in other studies [52, 58]. Participants' accounts suggest that recognition, acknowledgement, and identification of issues such as trauma, perfectionism, imposter syndrome, or neurodiversity may be important in improving support for GPs as human beings. This highlights a potential area where workplace policies could be considered to better support humane doctors.

In “GP wellbeing: combatting burnout in general practice” from 2017, Staten describes the personality factors that cause burnout as perfectionism, pessimism, need to control and reluctance to delegate, and being a high achiever [59]. Many of these personal factors are exactly what make a good GP. Often, the factors which make a good GP, such as empathy, which could be born from previous trauma, or perfectionism and attention to detail stemming from neurodiverse traits, could be the same factors that make an individual vulnerable. Rather than arguing for changed selection processes, we argue for greater understanding, support and accommodation of individual vulnerabilities and risk factors for burnout. The challenge from our PPI group member regarding the identification of personal factors that predispose to burnout may reflect wider cultural, press and systemic attitudes that neglect the human aspect of doctors. These wider attitudes towards doctors as fallible humans require further investigation. The human to human interaction in primary care is irreplaceable [60], and both doctor and patient deserve good health.

This study explores how GPs who have experience of burnout describe their own personal predisposing factors to their burnout. However these personal factors interacted with multiple other occupational and public factors to precipitate burnout. Further research is needed into the experiences and needs of neurodivergent GPs, those with past trauma or ACEs, and those with perfectionist traits or imposter syndrome, and how this interacts with the workplace, and it is essential to understand this for the future of recruitment and retention of GPs.

## Supplementary Material

cmag012_Supplementary_Data

## Data Availability

These data will be anonymized and may be available on reasonable request. Whitehead, Ishbel (2024). Depth Interviews with UK GP regarding Burnout and spiritual health. Newcastle University. Dataset. https://doi.org/10.25405/data.ncl.25639059.v1.
